# Movement Behaviors and the Role of Self‐Reported Symptoms and Well‐Being: A Dynamic Structural Equation Modeling Approach Among Head and Neck Cancer Patients

**DOI:** 10.1002/pon.70350

**Published:** 2025-11-29

**Authors:** Sabina Ulbricht, Antje Ullrich, Christina Grosse‐Thie, Daniel Strueder, Markus Blaurock, Chia‐Jung Busch, Christian Junghanss, Sabine Felser

**Affiliations:** ^1^ Department SHIP‐KEF Institute for Community Medicine University Medicine Greifswald Greifswald Germany; ^2^ Department of Medicine Clinic III, Hematology, Oncology Palliative Medicine Rostock University Medical Center Rostock Germany; ^3^ Oncology and Hematology Practice Rostock Germany; ^4^ Department of Otorhinolaryngology, Head and Neck Surgery Rostock University Medical Center Rostock Germany; ^5^ Department of Otorhinolaryngology, Head and Neck Surgery University Medicine Greifswald Greifswald Germany

**Keywords:** accelerometry, bidirectionality modeling, dynamic structural equation, head and neck cancer, physical activity, sedentary behavior

## Abstract

**Aim:**

This study aimed to identify individual variability in day‐to‐day physical activity (PA) and sedentary behavior (SB) as well as effects of pain, fatigue, and well‐being among patients with head and neck cancer (pwHNC) on both movement behaviors.

**Methods:**

A total of 52 pwHNC (age ≥ 18 years, participation rate 73.2%) consented to wear an accelerometer during waking hours for seven consecutive days and to answer questionnaires. Applying a predefined accelerometer protocol resulted in a final analysis sample of 41 pwHNC. A dynamic structural equation modeling approach (DSEM) was employed that combines time series modeling and multilevel modeling. *At the within‐person level*, autoregressive and bidirectional relationships between PA and SB were analyzed. *At the between‐person level*, moderator effects of pain, fatigue, and well‐being on these relationships were analyzed.

**Results:**

A proportion of 87.8% among the pwHNC were men, mean age 65.8 years, and 72.2% had finished curative care. The average accelerometer wear time was 710.2 ± 153.1 min/d. Of this time, an average of 461.3 ± 151.0 min/d was spent in SB and 248.9 ± 121.6 min/d in PA.

At the within‐person level, PA and SB data showed no significant autoregressive relationships; that is, previous PA (or SB) did not predict PA (or SB) on the next day, respectively. No bidirectional relationships were found between PA and SB; that is, previous PA did not predict the next‐day SB and vice versa. At the between‐person level, neither fatigue, pain nor well‐being significantly changed the slope and path parameters of PA and SB.

**Conclusion:**

This study serves as an example of how DSEM can be used to examine how different movement behaviors interact with each other. Presumably, there is a need for more long‐term assessment for the detection of consistency in the temporal information of movement behaviors in pwHNC.

**Trial Registration:**

German Registry of Clinical Trials (DRKS00028062)

## 1 Introduction

Head and neck malignancies account for 4.8% of all tumors and approximately 4.6% of all cancer‐related deaths [1]. Survivors often suffer from pain, cancer‐related fatigue, and other disabilities (e.g., dysphagia) that persist years after treatment [2, 3]. Occurrence of these symptoms is associated with reduced long‐term quality of life (QoL) [4]. Supervised exercise interventions can reduce cancer‐related symptoms such as pain and fatigue in the short term while improving QoL [5, 6]. However, chronic burden of symptoms often prevents physical activity (PA) in daily life [3, 7].

According to self‐reported data, less than 50% of cancer patients [8, 9] achieve the World Health Organization recommendations of 150 min moderate PA or 75 min vigorous PA a week including muscle‐strengthening activities at least 2 days a week [10, 11]. Among patients with head and neck cancer (pwHNC), the proportion who meet recommended PA levels are significant lower compared to breast or colorectal cancer patients (9% vs. 30% or 25%, respectively) [12, 13, 14]. As more information becomes available on the association between PA and mortality reduction in cancer patients [15], it seems important to consider the total volume of PA and not just specific activities performed at moderate‐to‐vigorous intensity. Within the last decade, sedentary behavior (SB) has become a relevant issue in cancer research [16]. Understanding the relationship between both movement behaviors as well as potential barriers to regular PA such as fatigue and pain, could be important to engage more PA.

Wearables such as accelerometers are crucial to capture the total volume of PA and SB [17]. They are widely used for objective measurement of movement behaviors in clinical and research settings [18]. Results are comprehensive data sets with repeated measurements in the same individual. However, analysis of temporal relationships of PA and SB in accelerometer data are often neglected [19]. Thus, estimating the autoregressive relations (= prediction of next‐day occasion by using previous occasion) and bidirectional day‐to‐day dynamic in both movement behaviors might be beneficial to explore variability on the intra‐individual level. The utility of this approach lies in the potential to intervene in one behavior, e. g., less SB while simultaneously increasing another one, e. g., PA [20]. Using an advanced statistical approach (= dynamic structural equation modeling; DSEM) [19, 21], we aimed to identify individual variations in day‐to‐day PA and SB among pwHNC, examine the relationship between both accelerometer‐based outcomes, and consider temporal information of the process [22]. To analyze the following research questions, a multilevel cross‐lagged model of the DSEM framework was applied:

1. Within‐person level: Does previous PA predict the next‐day PA and does previous SB predict the next‐day SB (= autoregressive relations)? In addition, does previous PA predict the next‐day SB and does previous SB predict the next‐day PA (= bidirectional relations)?
2. Between‐person level: Do cancer‐related symptoms such as pain, fatigue, and well‐being moderate these autoregressive and bidirectional relationships, as PA has been shown to reduce both symptoms while improving QoL of pwHNC.

## 2 Methods

### 2.1 Participants and Procedure

A total of 71 pwHNC, aged ≥ 18 years, were approached during outpatient consultations at a university hospital as well as during meetings of a self‐help group in the Northeast of Germany between January and September 2022. All were asked to wear a tri‐axial accelerometer (ActiGraph GT3X, Pensacola, FL, USA) for seven consecutive days, to fill in a self‐administered survey, and to provide consent for retrieval of specified clinical data from their medical record.

Those who agreed (*n* = 52 patients; 73.2%) provided their written informed consent. Participants were instructed to wear the device on their right hip during waking hours, except for water activities (e.g., showering or swimming). Further, a fact sheet comprising the wearing instructions was handed over. All participants received a voucher (25 Euro) upon mailing the device. The study protocol was approved by the Rostock University Medicine Ethics Committee (A2021‐0248) and registered with the German Registry of Clinical Trials (DRKS00028062). Withdrawal of written informed consent (*n* = 1), missing accelerometer data due to technical error during device initialization (*n* = 2), and a wear time of < 500 min per day (min/day), on less than 4 days a week including 1 weekend day (*n* = 8) (in accordance with Migueles [23]), resulted in a final analysis sample of 41 participants.

### 2.2 Measures

#### 2.2.1 Accelerometer‐Based Measures

Using ActiLife software version 6.12.0 (Actigraph Corp., Pensacola, USA), accelerations were recorded at a sampling frequency of 30 Hz in 10 s (sec) intervals. Raw data was downloaded, and 10 s data was collapsed to 60 s epochs; movement intensity was expressed as counts‐per‐minute (cpm). Data from the vertical axis was used. Non‐wear time was defined as time segments of ≥ 60 consecutive minutes of continuous zero activity counts, allowing for 2 min of counts between 0 and 100. Cutoffs for movement intensities according to threshold criteria were used: values < 100 cpm were defined as SB and values ≥ 100 cpm as PA. The mean daily time spent in PA and SB was calculated as total minutes of SB per day (min/d), and as total minutes of PA per day (min/d), respectively.

#### 2.2.2 Self‐Reported Survey Data

Sex (binary: female/male), age in years (continuous), current partnership (binary: yes/no), and school education (categorial: < 10 years/= 10 years/> 10 years) were assessed.

Smoking was assessed using the answer categories 1 = “daily”, 2 = “occasional”, 3 = “former”, and 4 = “never smoking”. A three‐stage variable was built by collapsing the categories 1 and 2 to the category “current smoking”. The frequency of alcohol consumption was assessed using the first item of the Alcohol Use Disorders Identification Test “How often do you have a drink containing alcohol?” [24]. Answer categories were 0 = “never”, 1 = “monthly”, 2 = “2 to 4 times a month”,3 = “2 to 4 times a week”, and 4 = “4 or more times a week”. A binary variable was built by collapsing the last three categories (no consumption/consumption).

Cancer‐related symptoms such as pain, fatigue, and well‐being were assessed using a continuous 11‐point numerical rating scale (NRS) ranged between 0 “none” to 10 “very strong” (=pain), 0 “not at all” to 10 “very often” (=fatigue), or 0 “very bad” to 10 “very good” (=well‐being) [25]. Dyspnea was assessed using the Modified Medical Research Council Dyspnea Scale (categorical: 0 = “not troubled with breathlessness except with strenuous exercise”, 1 = “troubled by shortness of breath when hurrying on the level or walking up a slight hill”, 2 = “walks slower than people of the same age on the level because of breathlessness or has to stop for breath when walking at own pace on the level”, 3 = “stops for breath after walking about 100 yards or after a few minutes on the level” and 4 = “too breathless to leave the house or breathless when dressing or undressing”) [26]. Due to the limited number of responses in the four categories of reported complaints (1–4), these categories were collapsed into one binary variable (no complaints/complaints). Body mass‐index (BMI) was calculated by dividing self‐reported body weight (in kilograms) by height (in meters) squared (kg/m^2^) (continuous). Survey information was gathered once, at the start of the study, and before the participants wore the accelerometer.

#### 2.2.3 Clinical Data From Medical Record

The following disease‐specific characteristics of participants were retrieved from the clinical medical records: tumor location (categorial: oropharynx/larynx/others), cancer stage (categorial: I to II/III to IV), time after initial diagnosis in months (continuous) as well as current treatment (categorical: curative care/aftercare/palliative care).

### 2.3 Statistical Analyses

#### 2.3.1 Study Participant Characteristics

Participant characteristics were described as mean (M) with standard deviation (SD) for continuous variables and as frequencies (*n*) and percentages (%) for categorical variable, respectively. Characteristics of study participants who met the defined criteria of accelerometer wear time (*n* = 41) were compared with those who did not (*n* = 8). Both patient groups did not differ in terms of sex (*p* = 0.585; Fisher's exact test) and age (*p* = 0.962; *t*‐test). The Spearman correlation was used to calculate the strength and direction of the relationship between PA and SB per day. Descriptive analysis was performed using STATA (StataCorp LLC, Texas, USA, Version: 18.0).

#### 2.3.2 Analysis Framework: Dynamic Structural Equation Modeling

**FIGURE 1:**
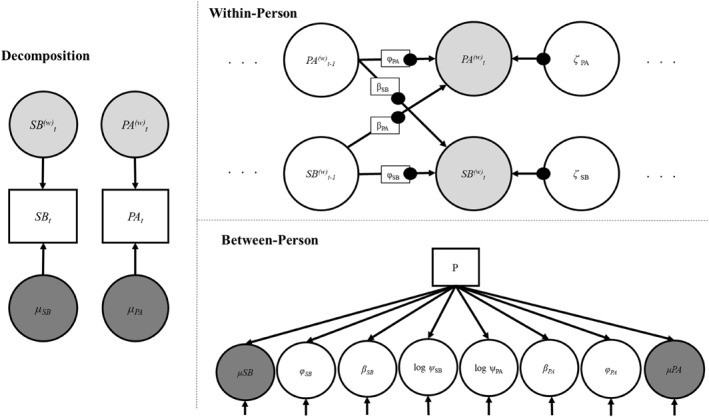
Schematic representation of the multi‐level time series dynamic structural equation model. The figure shows the decomposition of the data into within‐ and between‐person components, including an observed predictor for random effects at the between level. Black dots display random effects. Abbreviations. All components: SB = sedentary behavior, PA = physical activity. Decomposition: _t_ = time points, μ = between‐person means, ^(w)^ = within‐person estimates. Within‐person component: _t_ = time points, _t‐1_ = previous time points, *φ* = autoregressive estimates, *β* = bidirectional (cross‐lagged) estimates, *ζ* = within‐person, time‐specific residuals. Between‐person component: μ = between‐person means, *φ* = autoregressive estimates, *β* = bidirectional (cross‐lagged) estimates, *ψ* = dynamic errors, P = predictor.

This study uses an advanced statistical approach to examine the relationship between movement behaviors and survey data [27]. The objective assessment of movement behaviors results in data sets with a large number of repeated measurements of the same individual. DSEM can be used to model (i) data sets within and between individuals and (ii) dynamic (i.e., lagged) relationships between observed (and/or unobserved) variables over time by combining up to three traditional modeling approaches [19]. In this study, the model based on a combination of time series modeling and multilevel modeling. In general, the DSEM approach decomposes repeatedly measured observed variables (*x*, *y*) assessed at multiple time points (*t*) for different individuals (*i*) into time‐varying (level 1: within‐person) and time‐invariant (level 2: between‐person) components, respectively. DSEM decomposition extracts the individual specific means of each repeatedly measured movement behavior variable (*μx*, *μy*), which comprise the between components. Each individual's deviations from those specific means at each multiple time point [x_*t*_^*(w)*^, y_*t*_^*(w)*^] comprise the within components [19, 28]. Figure 1 depicts the model structure.

For both components, equations with estimates from the within‐person component (= random slopes) and expected scores for the random effects in the between‐person component can be specified. The intercepts in the equations indicate the expected scores when the included predictor is zero, while the regression coefficients indicate the degree to which random effects can be estimated from the observed predictor (*P*; see Supporting Information S1: Figure S1) [19, 29]. Pain, fatigue, and well‐being as between‐person predictor were grand‐mean centered and each were entered into three separate models to examine individual differences in the within‐person relationships between PA and SB, respectively [29]. Estimating whether the presence of each predictor significantly alter the slope and path parameters, help to decide whether moderated effects are present. Three separate models were run using Bayesian estimation techniques and Markov chain Monte Carlo chains algorithms with randomly generated seeds [29]. For the models, 50.000 iterations were run with a thin of 10. All Bayesian estimations were conducted using diffuse priors by default, which contain few preexisting information regarding parameter values [19, 30]. The models were applied using Mplus (Version 8.4; [31]).

## 3 Results

### 3.1 Sample Characteristics

As shown in Table 1, more men (87.8%; *n* = 36) than women (12.2%; *n* = 5) provided data for analysis; with a mean age of 65.8 years (SD = 9.6). Most study participants reported that they attended school for 10 years (58.6%; *n* = 24) and were currently living in a partnership (61.0%; *n* = 25). The proportion of never‐smokers was 12.2% (*n* = 5). More than half of the study participants reported alcohol consumption (56.3%; *n* = 22).

**TABLE 1:** Sample characteristics and reported symptoms (*n* = 41).

Characteristics	
Age, years	65.8 ± 9.6
Sex	
Men	36 (87.8)
Women	5 (12.2)
Partner	
Yes	25 (61.0)
No	16 (39.0)
School, years	
< 10	11 (26.8)
= 10	24 (58.6)
> 10	6 (14.6)
BMI, kg/m^2^	25.4 ± 5.0
Smoker	
Current	13 (31.7)
Former	23 (56.1)
Never	5 (12.2)
Alcohol consumption	
Yes	22 (53.6)
No	19 (46.3)
Tumor location	
Oropharynx	15 (36.6)
Larynx	9 (21.9)
Others	17 (41.5)
Current therapy phase	
Curative care	2 (4.9)
Aftercare	30 (72.2)
Palliative care	9 (21.9)
Cancer stage	9 (30.0)
I–II	21 (70.0)
III–IV	
Time after initial diagnosis, months	59.3 ± 53.0
Pain^a^	1.9 ± 2.4
Fatigue^a^	3.5 ± 3.4
Dyspnea	
No complaints	18 (43.9)
Complaints	23 (56.1)
Well‐being^a^	6.4 ± 2.4

*Note:* Participant characteristics were described as mean (*M*) with standard deviation (SD) for continuous variables and as frequencies (*n*) and percentages (%) for categorical variables.

Abbreviation: BMI, body mass‐index.

^a^ Pain, fatigue, and well‐being were assessed with an 11‐point numerical Rating Scale ranged between 0 “*not at all*” to 10 “*very often*” (fatigue), 0 “*none*” to 10 “*very strong*” (pain intensity), or 0 “*very bad*” to 10 “*very good*” (well‐being).

Participants were diagnosed with a tumor located in the oropharynx (36.6%; *n* = 15), in the larynx (21.9%; *n* = 9), or other areas (41.5%; *n* = 17) with a cancer stage of category III or IV (70.0%; *n* = 21) and a mean time of 59.3 months (SD = 53.0) after their initial tumor diagnosis.

Most of them had finished treatment with curative intend (72.2%; *n* = 30). Participants reported an average NRS score of 1.9 for pain (SD = 2.4), 3.5 for fatigue (SD = 3.4), and 6.4 for well‐being (SD = 2.4). In general, complaints with varying levels of dyspnea were present in the sample (56.1%; *n* = 23).

### 3.2 Accelerometry: Wear Time and Movement Behaviors

**FIGURE 2:**
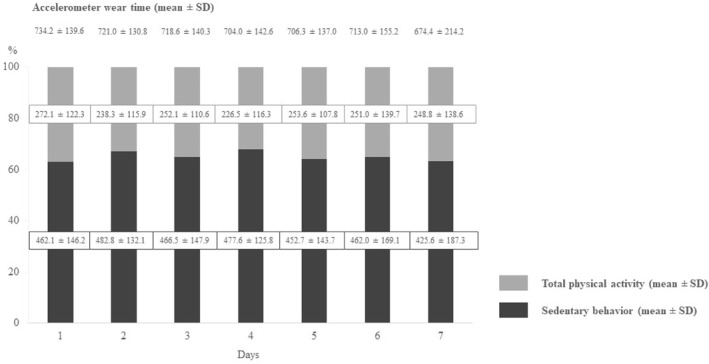
Results of average accelerometer wear time, total time spent in sedentary behavior and physical activity per day over the 7‐day accelerometer wear period among the study sample (*n* = 41). The dark gray segments represent the time spent in sedentary behavior and the light gray segments represent the time spent in physical activity. SD = standard deviation.

The average accelerometer wear time was 710.2 ± 153.1 min/d (minimum on day seven: 674.4 ± 214.2 min/d, maximum on day one: 734.2 ± 139.6 min per day). Of this time, an average of 461.3 ± 151.0 min/d was spent in SB (minimum on day seven: 425.6 ± 187.3 min/d, maximum on day two: 482.8 ± 132.1 min/d), representing 64.7% (minimum on day seven: 62.4%, maximum on day four: 68.3%). In contrast, 248.9 ± 121.6 min/d were spent in PA (minimum on day four: 226.5 ± 116.3 min/d, maximum on day one: 272.1 ± 122.3 min/d), corresponding to 35.0% (minimum on day four: 31.7%, maximum on day one: 37.1%) (Figure 2). The correlations between SB and total PA per day varied between *r* = −0.14 (day 7) and *r* = −0.56 (day 6); the *p*‐values were less than 0.05, except on day 4 and 7 (*p* = 0.169 and *p* = 0.376, respectively).

### 3.3 Self‐Reported Symptoms and Well‐Being

**FIGURE 3:**
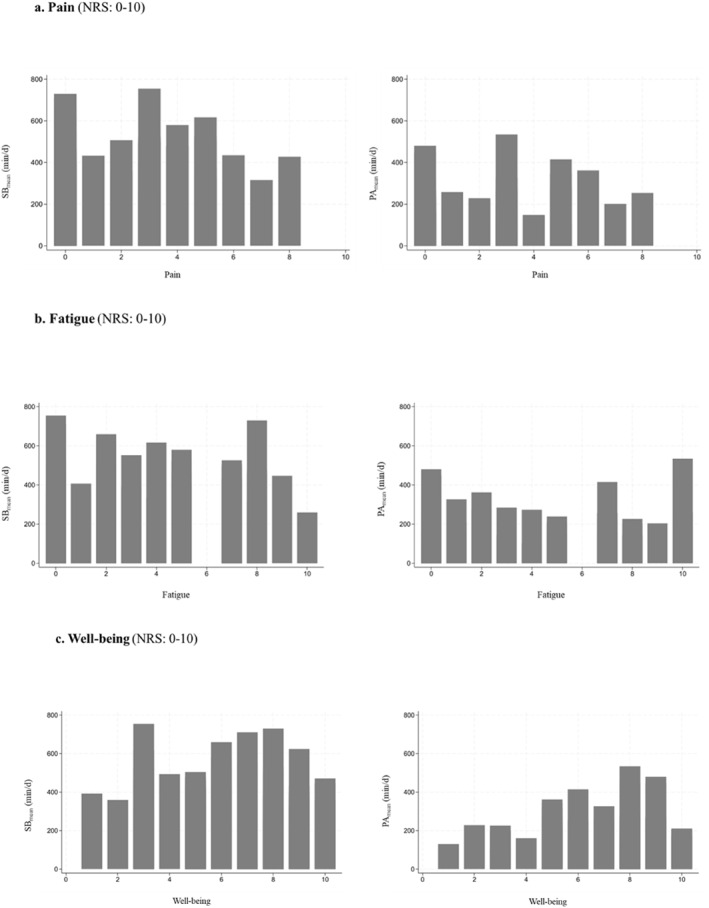
Results on the relationships between time spent in accelerometer‐based sedentary behavior and physical activity and pain, fatigue, and well‐being in the study sample. Pain (a), fatigue (b), and well‐being (c) were assessed with an 11‐point numerical Rating Scale (NRS) ranged between 0 “*none*” to 10 “*very strong*” (pain), 0 “*not at all*” to 10 “*very often*” (fatigue), or 0 “*very bad*” to 10 “*very good*” (well‐being). min/d = minutes per day, NRS = numeric Rating Scale, PA = physical activity, SB = sedentary behavior.

Figure 3 shows the descriptive relationships between the time spent in PA, SB and the responses of the items on self‐reported symptoms (pain, fatigue) and well‐being.

### 3.4 DSEM Estimates: Autoregression, Bidirectionality, and Between‐Person Predictor Relationship

Table 2 summarizes the results of the unstandardized and standardized parameter estimates of the three models (model 1: pain as between‐person predictor; model 2: fatigue as between‐person predictor; model 3: well‐being as between‐person predictor). The models explained 21% (model 1) to 22% (model 2/model 3) of the within‐person variability in SB and 13% (model 3), 14% (model 1), or 15% (model 2) of the within‐person variability in PA, respectively.

**TABLE 2:** Results of multilevel time series dynamic structural equation models (*n* = 41).

	Model 1: Pain		Model 2: Fatigue		Model 3: Well‐being	
Path symbols^a^	Estimate	95% credibility interval	Estimate	95% credibility interval	Estimate	95% credibility interval
Unstandardized parameter estimates						
Fixed effects						
*μ*_SB_	460.24	[418.95, 503.59]^b^	462.67	[420.24, 505.86]^b^	460.56	[417.99, 503.52]^b^
*μ*_PA_	244.43	[209.54, 280.00]^b^	243.80	[209.41, 280.45]^b^	245.32	[213.10, 277.44]^b^
*φ*_SB_	0.18	[−0.02, 0.40]	0.18	[−0.02, 0.38]	0.18	[−0.02, 0.40]
*φ*_PA_	0.04	[−0.16, 0.27]	0.06	[−0.14, 0.31]	0.05	[−0.15, 0.28]
*β*_SB_	−0.00	[−0.34, 0.30]	−0.01	[−0.32, 0.28]	−0.02	[−0.35, 0.29]
*β*_PA_	−0.01	[−0.10, 0.08]	−0.02	[−0.11, 0.08]	−0.02	[−0.11, 0.08]
log *ψ*_SB_	9.10	[8.83, 9.35]^b^	9.08	[8.81, 9.35]^b^	9.09	[8.79, 9.36]^b^
log *ψ*_PA_	8.11	[7.71, 8.49]^b^	8.13	[7.72, 8.51]^b^	8.12	[7.73, 8.49]^b^
Random‐effects variances						
*μ*_SB_	13,875.64	[6215.59, 26,248.31]^b^	13,569.17	[6528.31, 25,417.60]^b^	13,497.20	[6141.28, 25,766.39]^b^
*μ*_PA_	10,851.08	[5591.17, 18,763.23]^b^	10,482.78	[5130.90, 18,962.13]^b^	9839.28	[5080.12, 17,389.47]^b^
*φ*_SB_	0.07	[0.01, 0.22]^b^	0.06	[0.00, 0.22]^b^	0.06	[0.00, 0.22]^b^
*φ*_PA_	0.06	[0.00, 0.27]^b^	0.07	[0.00, 0.28]^b^	0.06	[0.00, 0.26]^b^
*β*_SB_	0.22	[0.02, 0.79]^b^	0.15	[0.01, 0.68]^b^	0.26	[0.03, 0.87]^b^
*β*_PA_	0.01	[0.00, 0.04]^b^	0.01	[0.00, 0.04]^b^	0.01	[0.00, 0.04]^b^
log *ψ*_SB_	0.19	[0.01, 0.58]^b^	0.22	[0.04, 0.60]^b^	0.21	[0.03, 0.66]^b^
log *ψ*_PA_	1.03	[0.51, 1.94]^b^	1.02	[0.51, 1.95]^b^	1.00	[0.50, 1.95]^b^
Standardized parameter estimates						
Level: Within‐person (averaged across clusters)						
*φ*_SB_	0.17	[−0.00, 0.37]	0.18	[0.01, 0.35]^b^	0.17	[−0.01, 0.36]
*φ*_PA_	0.05	[−0.15, 0.24]	0.07	[−0.18, 0.28]	0.05	[−0.12, 0.27]
*β*_SB_	0.01	[−0.13, 0.15]	0.01	[−0.15, 0.16]	0.00	[−0.14, 0.15]
*β*_PA_	−0.02	[−0.17, 0.12]	−0.04	[−0.18, 0.11]	−0.03	[−0.18, 0.13]
*ζ*_SB_	0.79	[0.64, 0.92]^b^	0.78^b^	[0.64, 0.91]^b^	0.78	[0.63, 0.91]^b^
*ζ*_PA_	0.86	[0.70, 0.96]^b^	0.85^b^	[0.69, 0.95]^b^	0.87	[0.71, 0.96]^b^
Level: Between‐person						
*φ*_SB_ ON predictor	0.14	[−0.39, 0.74]	0.25	[−0.34, 0.81]	0.22	[−0.34, 0.84]
*φ*_PA_ ON predictor	−0.16	[−0.79, 0.48]	−0.12	[−0.81, 0.45]	0.08	[−0.60, 0.73]
*β*_SB_ ON predictor	−0.17	[−0.74, 0.27]	−0.34	[−0.92, 0.11]	0.03	[−0.42, 0.53]
*β*_PA_ ON predictor	−0.05	[−0.75, 0.78]	−0.30	[−0.93, 0.47]	0.19	[−0.57, 0.89]
log *ψ*_SB_ ON predictor	0.17	[−0.25, 0.74]	0.12	[−0.27, 0.60]	−0.06	[−0.54, 0.30]
log *ψ*_PA_ ON predictor	0.09	[−0.19, 0.34]	0.07	[−0.18, 0.32]	0.16	[−0.11, 0.40]
*μ*_SB_ ON predictor	−0.03	[−0.28, 0.24]	−0.03	[−0.29, 0.26]	−0.09	[−0.33, 0.16]
*μ*_PA_ ON predictor	0.06	[−0.17, 0.29]	−0.07	[−0.33, 0.18]	0.22	[−0.01, 0.43]
*σ μ*_PA_ μ_SB_	−0.56	[−0.80, −0.21]^b^	−0.56	[−0.81, −0.20]^b^	−0.55	[−0.80, −0.16]^b^
*R* ^*2*^						
Within‐level (averaged across clusters)						
SB	0.21	[0.08, 0.36]	0.22	[0.10, 0.36]	0.22	[0.09, 0.37]
PA	0.14	[0.04, 0.30]	0.15	[0.05, 0.31]	0.13	[0.04, 0.29]
Between‐level						
SB	0.01	[0.00, 0.09]	0.01	[0.00, 0.10]	0.01	[0.00, 0.11]
PA	0.01	[0.00, 0.08]	0.01	[0.00, 0.11]	0.05	[0.00, 0.18]

*Note:* Model 1 shows the results for pain, model 2 shows the results for fatigue, and model 3 shows the results for well‐being.

Abbreviations: PA, physical activity; SB, sedentary behavior; *µ*, between‐person means; *φ*, autoregressive estimates; *β*, bidirectional (cross‐lagged) estimates; *ψ*, dynamic errors; *ζ*, within‐person, time‐specific residuals; *σ*, covariance.

^a^ Path symbols correspond to the symbols used in Figure 1.

^b^ Significance based on 95% credibility interval.

Analyzes of the standardized within‐person coefficients (averaged across clusters) revealed almost no significant autoregressive relationships; that is, previous SB did not predict next‐day SB or previous PA did not predict next‐day PA. The standardized results of the within‐person level in model 2, showed a significant autoregressive relationship of SB (*Estimate* = 0.18, *p* = 0.022).

However, looking at the absolute standardized autoregressive coefficients of both movement behaviors for each individual, variability in values were present in all three models (model 1: range of *φ*_*SB*_ = −0.10 to 0.43, range of *φ*_*PA*_ = −0.23 to 0.22; model 2: range of *φ*_*SB*_ = −0.08 to 0.51, range of *φ*_*PA*_ = −0.20 to 0.29; model 3: range of *φ*_*SB*_ = −0.05 to 0.41, range of *φ*_*PA*_ = −0.13–0.24). See Supporting Information S1 Figure S2 for the full results. An autoregressive value close to zero means that an individual will rapidly returns to a typical amount of time spent in PA or SB (= strong attraction dynamic), while a value close to one means that consecutive days of high or low PA or SB were followed by values above or below their typical amount of time spent in these behaviors [20]. The average autoregressive values in this study were positive for PA and SB for all models (range of 0.05–0.07 for PA; range of 0.17–0.18 for SB) and closer to zero than one (i.e., strong attractor). Negative autoregressive values imply a reflexive back‐and‐forth shift between the values of an individual's typical PA or SB level; for example, if an individual spends less than their typical amount of time in SB on one day, there are more likely to become more sedentary the next day (=antipersistance) [32]. For SB, 4.9% (model 2 and 3) to 9.8% individuals (model 1) had an autoregressive value below zero, reflecting a low level of antipersistance. In PA, the proportion of absolute negative autoregressive values was higher (model 1: 39.0%; model 2 and 3: 29.3%); thus, there was a higher level of antipersistance in the study sample.

In all three models examining the bidirectional relationships between both movement behaviors, no significant standardized cross‐lagged effects were found [see Table 2; model 1: Deviance (DIC) = 6701.61, Estimated Number of Parameters (*pD*) = 138.05; model 2: *DIC* = 6704.49, *pD* = 134.91; model 3: *DIC* = 6707.80, *pD* = 142.82]. The absolute standardized cross‐lagged *coefficients* of both movement behaviors for each individual showed variability in all three models (see Supporting Information S1 Figure S2; model 1: range of *β*_*SB*_ = −0.65 to 0.40, range of *β*_*PA*_ = −0.13 to 0.03; model 2: range of *β*_*SB*_ = −0.86 to 0.42, range of *β*_*PA*_ = −0.14 to 0.08; model 3: range of *β*_*SB*_ = −0.81 to 0.54, range of *β*_*PA*_ = −0.14 to 0.06). In addition, a significant negative covariance between SB and PA (*σ μ*_PA_ μ_SB_) was found at the between‐person level (model 1: *Estimate*: −0.56, *p* = 0.002; model 2: *Estimate*: −0.56, *p* = 0.003; model 3: *Estimate*: −0.55, *p* = 0.003).

Additionally, none of the between‐person predictors significantly changed the slope and path parameters of the movement behaviors. Thus, no significant standardized effects were found in model 1 (*β*_SB_ ON pain *Estimate* = −0.17, *p* = 0.236; *β*_PA_ ON pain *Estimate* = −0.05, *p* = 0.443), model 2 (*β*_SB_ ON fatigue *Estimate* = −0.36, *p* = 0.07; *β*_PA_ ON fatigue *Estimate* = −0.30, *p* = 0.209), and model 3 (*β*_SB_ ON well‐being *Estimate* = 0.03, *p* = 0.444; *β*_PA_ ON well‐being *Estimate* = 0.19, *p* = 0.286).

## 4 Discussion

The present study consists of a comprehensive investigation of individual variations in day‐to‐day PA and SB over 7 days in a sample of pwHNC, most of them in aftercare, by using a within‐person perspective as well as a between‐person perspective. Our study found three main results: First, PA and SB data showed no significant autoregressive relationships from one day to the next; that is, previous SB did not predict SB next day and previous PA did not predict PA next day. Second, no bidirectional relationships were found between PA and SB at the within‐person level; that is, previous PA did not predict the next‐day SB and vice versa. Third, neither fatigue nor pain or well‐being significantly changed the slope and path parameters of PA and SB.

This is the first study using a statistical framework for modeling within‐person parameters to account for temporal information in movement behavior such as the autoregressive association from SB one day to SB the next day (and from PA one day to PA the next day) and the bidirectional cross‐lagged association from SB one day to PA next day, and vice versa in pwHNC. In contrast to accelerometer‐based studies using the DSEM framework in children, adolescents, and adults [20, 27, 33, 34], the consistency of both movement behaviors over the seven consecutive days is not very pronounced in our sample. This is shown by the non‐significant autoregressive values. However, these are a prerequisite for a “set point” (i.e., a typical PA or SB level) around which upward or downward deviations can be identified over a period [20]. Presumably, treatment or post‐treatment cancer‐related symptoms contribute to difficulties in performing consistent PA and SB activities. This assumption is supported by results from studies in pwHNC showing that restrictions in functional issues, e. g., swallowing and deconditioning are associated with lower QoL [35, 36].

Despite the variability in tumor types and subsites in head and neck cancer, non‐surgical treatment is standardized with limited stratification based on these factors. This relatively uniform therapeutic approach contributes to the development of side effects, including chronic pain and fatigue. The importance of these issues in pwHNC, persisting even up to 5 years post‐diagnosis, has been highlighted by Howren [37] and Berg [38]. Approximately one third of pwHNC experienced at least some pain with 17.5% indicating moderate or severe pain at 5 years post‐diagnosis [37]. The proportion of patients defined as having “high” physical, emotional, or cognitive fatigue at 5 years post‐diagnosis were 16%, 19%, or 13% [38].

The absence of moderating effects of chronic pain and fatigue on bidirectional cross‐lagged associations between SB and PA in our study may be related to the low average of these symptoms (*M*_pain_ = 1.9 and *M*_fatigue_ = 3.5) on an 11‐point NRS ranged between 0 and 10 in the sample. A previous published study indicates low symptom burden and high QoL among patients with large head and neck squamous cell cancer [39]. The authors discussed this phenomenon in the light of higher prevalences in risk behaviors such as smoking and alcohol consumption in pwHNC samples from Central European countries compared with pwHNC samples from the USA [39]. The proportion of pwHNC with a history of smoking in our study was 80%, more than half reported current alcohol consumption. In addition, pwHNC with low educational level represent a quarter of our sample. The question arises whether pwHNC caused by smoking and alcohol consumption may have lower expectations regarding their own bodily function compared to those with a healthier lifestyle and HPV‐associated tumors [39]. Consequently, patients with substance use associated tumors might report lower symptom scores and a higher subjective QoL.

Another potential explanation for the absence of within‐associations between PA and cancer‐related symptoms may be the sample cohort. It can be hypothesized that some of the participants may have been highly motivated and health‐conscious, a factor which led them to maintain their PA levels largely independently of temporary changes in their symptoms. This adaptive or compensatory behavior may result in a weakening of the correlation between PA and cancer‐related symptoms. It can be hypothesized that habitual PA patterns and daily routines may be able to exert a greater influence on well‐being than day‐to‐day variations.

The utilization of instruments for the assessment of fatigue or pain with a cut‐off value would have facilitated a more comprehensive and multidimensional characterization of the symptom burden. Nevertheless, we opted for short NRS with a single item in order to minimize the burden on participants with the aim of achieving a higher participation rate. This approach ensured the collection of complete data on core variables. However, the predictive performance of chronic pain and fatigue might be improved by recording them several times a day along accelerometry. By using methods such as ecological momentary assessments (EMA) to collect data on cancer‐related symptoms, we could quantify the impact of pain or fatigue at the same time as PA and SB, immediately afterward and the next day. In addition, other physical conditions (e.g., side effects of medication), environmental factors (e.g., housing conditions), and social aspects (e.g., movement behavior of the partner or other relatives such as children), may have a stronger association with PA and SB and should be considered in future studies too.

Furthermore, future research may benefit from the utilization of Artificial Intelligence (AI) supported, adaptive measurement in order to address some methodological limitations that have been identified in our study. To illustrate this point, one may consider the potential of wearable technology and mobile applications to facilitate the automated and context‐sensitive recording of PA and symptoms, either on a daily basis or throughout the day. Should the system identify a marked decline in PA, queries may be initiated to ascertain the potential causes underlying such a decline, including instances of fatigue or pain. Consequently, dynamic interactions between movement behavior and symptom burden could be characterized with greater precision, and personalized recommendations could be derived to support movement behavior depending on the situation and promote self‐regulation in everyday life. Therefore, the integration of AI and wearable technology data represents a significant potential for future research [40]. As research in this field continues to advance, there are three aspects that need to be considered. Firstly, it is important to understand why patients refuse to wear wearables. Secondly, the development of appropriate criteria for adherence to wearing regimes is essential. The third point to consider is how to protect personal data when using wearables in research and clinical settings.

### 4.1 Limitations

With a proportion of 73%, approximately 2 thirds of the eligible pwHNC consented to take part in the study, indicating a strong willingness within this patient group to engage with accelerometer‐based assessments. Nonetheless, no data were collected on individuals who have refused to participate in this study, which limits our ability to evaluate how representative the final sample is of the broader pwHNC population.

Women are underrepresented in our sample, accounting for 12.2%, despite men being up to 3.5 times more likely to develop head and neck cancer. The sample was higher aged, with a mean of 67 years and a standard deviation of 9.6. The distribution of sex, age, and also the exclusion of participants who did not meet the accelerometer wear criteria may have contributed to the selection. In addition to the sample characteristics and accelerometer data handling, the results were based on a small sample of pwHNC. A recently published review addresses the issue of the small number of participants (ranging from two to 43) in studies testing wearables in pwHNC [41]. Additionally, our sample was limited to German‐speaking individuals, which may have excluded individuals with a migration background. Overall, this can lead to a lower generalizability of the results to the entire patient group.

The decision to use the NRS scale to measure fatigue, pain and well‐being instead of more comprehensive measures (e.g., the Multidimensional Fatigue Scale) may limit the interpretability of the null symptom findings. Further, gaining a more comprehensive understanding of how different movement behaviors interact may require longer measurement periods to capture the consistency of temporal patterns in PA and SB.

### 4.2 Clinical Implications

Monitoring compliance for a healthy lifestyle is a key challenge, particularly in pwHNC where regular PA is limited. A better understanding of the bidirectional day‐to‐day dynamic in PA and SB may facilitate to identify the “teachable moment” whereby pwHNC can make positive changes in both movement behaviors. Considering the role of co‐occurring factors, such as pain and fatigue, in these daily dynamics could offer a more comprehensive understanding of the unique challenges faced by pwHNC allowing healthcare professionals to recommend the most suitable intervention for each patient.

### 4.3 Conclusion

This study represents one of the first efforts to explore the temporal dynamics ‐ specifically autoregressive and bidirectional relationships—between PA and SB in pwHNC. Although no significant associations were identified, these findings underscore the need for future research with larger sample sizes to enhance statistical power and detect potential patterns.

## Funding

The study was supported by the Northern German Universities Association (VNU) as part of the “Impulse Research” program (VNU Impulse #60).

## Ethics Statement

Rostock University Medicine Ethics Committee (A2021‐0248).

## Consent

All participants provided written informed consent.

## Conflicts of Interest

The authors declare that the research was conducted in the absence of any commercial or financial relationships that could be construed as a potential conflict of interest.

## Supporting information


Supporting Information S1

## Data Availability

The data are available on request.

## References

[1] H. Sung , J. Ferlay , R. L. Siegel , et al., “Global Cancer Statistics 2020: GLOBOCAN Estimates of Incidence and Mortality Worldwide for 36 Cancers in 185 Countries,” CA: A Cancer Journal for Clinicians 71, no. 3 (2021): 209–249, 10.3322/caac.21660. 33538338

[2] R. Ordonez , A. Otero , I. Jerez , J. A. Medina , Y. Lupianez‐Perez , and J. Gomez‐Millan , “Role of Radiotherapy in the Treatment of Metastatic Head and Neck Cancer,” OncoTargets and Therapy 12 (2019): 677–683, 10.2147/OTT.S181697. 30705596 PMC6343506

[3] S. Van Dijck , A. De Groef , J. Kothari , et al., “Barriers and Facilitators to Physical Activity in Cancer Survivors With Pain: A Systematic Review,” Supportive Care in Cancer 31, no. 12 (2023): 668, 10.1007/s00520-023-08141-3. 37922014

[4] A. Mehnert , J. Barth , M. Gaspar , et al., “Predictors of Early Retirement After Cancer rehabilitation‐A Longitudinal Study,” European Journal of Cancer Care 26, no. 5 (2017): e12528, 10.1111/ecc.12528. 27334307

[5] S. B. Al‐Mhanna , W. S. Wan Ghazali , M. Mohamed , et al., “Effectiveness of Physical Activity on Immunity Markers and Quality of Life in Cancer Patient: A Systematic Review,” PeerJ 10 (2022): e13664, 10.7717/peerj.13664. 35935260 PMC9354736

[6] J. Nakano , K. Hashizume , T. Fukushima , et al., “Effects of Aerobic and Resistance Exercises on Physical Symptoms in Cancer Patients: A Meta‐Analysis,” Integrative Cancer Therapies 17, no. 4 (2018): 1048–1058, 10.1177/1534735418807555. 30352523 PMC6247562

[7] C. Torregrosa , F. Chorin , E. E. M. Beltran , C. Neuzillet , and V. Cardot‐Ruffino , “Physical Activity as the Best Supportive Care in Cancer: The Clinician's and the Researcher's Perspectives,” Cancers (Basel) 14, no. 21 (2022): 5402, 10.3390/cancers14215402. 36358820 PMC9655932

[8] C. Mason , C. M. Alfano , A. W. Smith , et al., “Long‐Term Physical Activity Trends in Breast Cancer Survivors,” Cancer Epidemiology, Biomarkers & Prevention 22, no. 6 (2013): 1153–1161, 10.1158/1055-9965.EPI-13-0141. PMC3688258 23576689

[9] A. N. Troeschel , C. R. Leach , K. Shuval , K. D. Stein , and A. V. Patel , “Physical Activity in Cancer Survivors During ‘Re‐Entry’ Following Cancer Treatment,” Preventing Chronic Disease 15 (2018): E65, 10.5888/pcd15.170277. 29806579 PMC5985854

[10] P. Cormie , M. Atkinson , L. Bucci , et al., “Clinical Oncology Society of Australia Position Statement on Exercise in Cancer Care,” Medical Journal of Australia 209, no. 4 (2018): 184–187, 10.5694/mja18.00199. 29719196

[11] C. L. Rock , C. A. Thomson , K. R. Sullivan , et al., “American Cancer Society Nutrition and Physical Activity Guideline for Cancer Survivors,” CA: A Cancer Journal for Clinicians 72, no. 3 (2022): 230–262, 10.3322/caac.21719. 35294043

[12] C. Grimmett , J. Bridgewater , A. Steptoe , and J. Wardle , “Lifestyle and Quality of Life in Colorectal Cancer Survivors,” Quality of Life Research 20, no. 8 (2011): 1237–1245, 10.1007/s11136-011-9855-1. 21286822

[13] S. A. Huneidi , N. C. Wright , A. Atkinson , S. Bhatia , and P. Singh , “Factors Associated With Physical Inactivity in Adult Breast Cancer survivors‐A Population‐Based Study,” Cancer Medicine 7, no. 12 (2018): 6331–6339, 10.1002/cam4.1847. 30358141 PMC6308073

[14] L. Q. Rogers , K. S. Courneya , K. T. Robbins , et al., “Physical Activity and Quality of Life in Head and Neck Cancer Survivors,” Supportive Care in Cancer 14, no. 10 (2006): 1012–1019, 10.1007/s00520-006-0044-7. 16538497

[15] Y. Wang , H. Song , Y. Yin , and L. Feng , “Cancer Survivors could Get Survival Benefits From Postdiagnosis Physical Activity: A Meta‐Analysis,” Evidence‐Based Complementary and Alternative Medicine 2019 (2019): 1940903–1940910, 10.1155/2019/1940903. 31772591 PMC6854247

[16] K. M. Thraen‐Borowski , K. P. Gennuso , and L. Cadmus‐Bertram , “Accelerometer‐Derived Physical Activity and Sedentary Time by Cancer Type in the United States,” PLoS One 12, no. 8 (2017): e0182554, 10.1371/journal.pone.0182554. 28806753 PMC5555687

[17] D. S. Ward , K. R. Evenson , A. Vaughn , A. B. Rodgers , and R. P. Troiano , “Accelerometer Use in Physical Activity: Best Practices and Research Recommendations,” supplement, Medicine & Science in Sports & Exercise 37, no. S11 (2005): S582–S588, 10.1249/01.mss.0000185292.71933.91. 16294121

[18] H. Sievanen and U. M. Kujala , “Accelerometry‐Simple, but Challenging,” Scandinavian Journal of Medicine & Science in Sports 27, no. 6 (2017): 574–578, 10.1111/sms.12887. 28466474

[19] E. L. A. T. Hamaker and B. Muthén , “Dynamic Structural Equation Modeling as a Combination of Time Series Modeling, Multilevel Modeling, and Structural Equation Modelling,” in The Handbook of Structural Equation Modeling. R. H. Hoyle , ed. 2nd ed. (Guilford Press, 2021).

[20] B. Armstrong , L. B. Covington , G. J. Unick , and M. M. Black , “Featured Article: Bidirectional Effects of Sleep and Sedentary Behavior Among Toddlers: A Dynamic Multilevel Modeling Approach,” Journal of Pediatric Psychology 44, no. 3 (2019): 275–285, 10.1093/jpepsy/jsy089. 30476202 PMC6551589

[21] E. L. A. T. Hamaker and B. Muthén , “Supplementary Materials for DSEM Book Chapter,” (2012), https://ellenhamaker.github.io/DSEM‐book‐chapter/.

[22] N. K. Schuurman , E. Ferrer , M. de Boer‐Sonnenschein , and E. L. Hamaker , “How to Compare Cross‐Lagged Associations in a Multilevel Autoregressive Model,” Psychological Methods 21, no. 2 (2016): 206–221, 10.1037/met0000062. 27045851

[23] J. H. Migueles , C. Cadenas‐Sanchez , U. Ekelund , et al., “Accelerometer Data Collection and Processing Criteria to Assess Physical Activity and Other Outcomes: A Systematic Review and Practical Considerations,” Sports Medicine 47, no. 9 (2017): 1821–1845, 10.1007/s40279-017-0716-0. 28303543 PMC6231536

[24] T. F. Babor , J. C. Higgins‐Biddle , J. B. Saunders , and M. G. Monteiro , AUDIT: The Alcohol Use Disorders Identification Test: Guidelines for Use in Primary Health Care, 2nd ed. (World Health Organization, 2001), https://iris.who.int/handle/10665/67205.

[25] J. S. Temel , W. F. Pirl , C. J. Recklitis , B. Cashvally , and T. J. Lnych , “Feasibility and Validity of a one‐item Fatigue Screen in a Thoracic Oncology Clinic,” Journal of Thoracic Oncology 1, no. 5 (2006): 454–459, 10.1016/S1556-0864(15)31611-7. 17409899

[26] D. A. Mahler and C. K. Wells , “Evaluation of Clinical Methods for Rating Dyspnea,” Chest 93, no. 3 (1988): 580–586, 10.1378/chest.93.3.580. 3342669

[27] H. Bedree , S. A. Miller , J. Buscemi , R. N. Greenley , and S. T. Tran , “Using Technology to Assess Bidirectionality Between Daily Pain and Physical Activity: The Role of Marginalization During Emerging Adulthood,” Children 8, no. 9 (2021): 756, 10.3390/children8090756. 34572188 PMC8472665

[28] L. G. Speyer , A. L. Murray , and R. Kievit , “Investigating Moderation Effects at the Within‐Person Level Using Intensive Longitudinal Data: A Two‐Level Dynamic Structural Equation Modelling Approach in Mplus,” Multivariate Behavioral Research 59, no. 3 (2024): 620–637, 10.1080/00273171.2023.2288575. 38356288

[29] T. Asparouhov , E. L. Hamaker , and B. Muthén , “Dynamic Structural Equation Models,” Structural Equation Modeling 25, no. 3 (2018): 359–388, 10.1080/10705511.2017.1406803. 29624092

[30] B. Muthén and T. Asparouhov , “Bayesian Structural Equation Modeling: A More Flexible Representation of Substantive Theory,” Psychological Methods 17, no. 3 (2012): 313–355, 10.1037/a0026802. 22962886

[31] L. K. Muthén and B. O. Muthén , Mplus User’s Guide, 8th ed. In M. M . (1998–2017). (Ed.).

[32] S. De Haan‐Rietdijk , J. M. Gottman , C. S. Bergeman , and E. L. Hamaker , “Get over it! A Multilevel Threshold Autoregressive Model for State‐dependent Affect Regulation,” Psychometrika 81, no. 1 (2016): 217–241, 10.1007/s11336-014-9417-x. 25091047 PMC4764683

[33] B. Armstrong , M. W. Beets , A. Starrett , et al., “Dynamics of Sleep, Sedentary Behavior, and Moderate‐to‐Vigorous Physical Activity on School Versus Nonschool Days,” Sleep 44, no. 2 (2021): zsaa174, 10.1093/sleep/zsaa174. 32893864 PMC7982135

[34] E. A. Pyatak , D. Spruijt‐Metz , S. Schneider , et al., “Impact of Overnight Glucose on Next‐Day Functioning in Adults With Type 1 Diabetes: An Exploratory Intensive Longitudinal Study,” Diabetes Care 46, no. 7 (2023): 1345–1353, 10.2337/dc22-2008. 36862940 PMC10300522

[35] D. Adkins , T. Howard , A. Mangino , A. Phuong , A. Kejner , and N. Gupta , “Factors Influencing Quality of Life and Functional Status in Head and Neck Cancer Patients,” American Journal of Otolaryngology 45, no. 5 (2024): 104398, 10.1016/j.amjoto.2024.104398. 39068817

[36] J. T. Cheng , M. Ramos Emos , V. Leite , et al., “Rehabilitation Interventions in Head and Neck Cancer: A Scoping Review,” supplement, American Journal of Physical Medicine & Rehabilitation 103, no. 3S Suppl 1 (2024): S62–S71, 10.1097/PHM.0000000000002384. 38364033

[37] M. B. Howren , A. J. Christensen , and N. A. Pagedar , “Prevalence of Pain in a Sample of Long‐Term Survivors of Head and Neck Cancer,” American Journal of Otolaryngology 45, no. 4 (2024): 104300, 10.1016/j.amjoto.2024.104300. 38640810 PMC11168893

[38] M. Berg , E. Silander , M. Bove , L. Johansson , J. Nyman , and E. Hammerlid , “Fatigue in Long‐Term Head and Neck Cancer Survivors From Diagnosis Until Five Years After Treatment,” Laryngoscope 133, no. 9 (2023): 2211–2221, 10.1002/lary.30534. 36695154

[39] D. Struder , J. Ebert , F. Kalle , et al., “Head and Neck Cancer: A Study on the Complex Relationship Between QoL and Swallowing Function,” Current Oncology 30, no. 12 (2023): 10336–10350, 10.3390/curroncol30120753. 38132387 PMC10742452

[40] M. Birla , R. Rajan , P. G. Roy , I. Gupta , and P. S. Malik , “Integrating Artificial Intelligence‐Driven Wearable Technology in Oncology decision‐Making: A Narrative Review,” Oncology 103, no. 1 (2025): 69–82, 10.1159/000540494. 39072365 PMC11731833

[41] M. Shammas‐Toma , G. Sampieri , M. Xie , et al., “Wearable Technologies in Head and Neck Oncology:Scoping Review,” JMIR mHealth and uHealth 13 (2025): e72372, 10.2196/72372. 41071984 PMC12513687

