# Supplementary material for: Movement Behaviors and the Role of Self‐Reported Symptoms and Well‐Being: A Dynamic Structural Equation Modeling Approach Among Head and Neck Cancer Patients
Source: Psychooncology. 2025 Nov 29;34(12):e70350. doi: 10.1002/pon.70350 (PMC12664338; doi:10.1002/pon.70350)
Supplement: Supplementary file 1 — Supporting Information S1 [file PON-34-e70350-s001.docx]

**Supplement material**


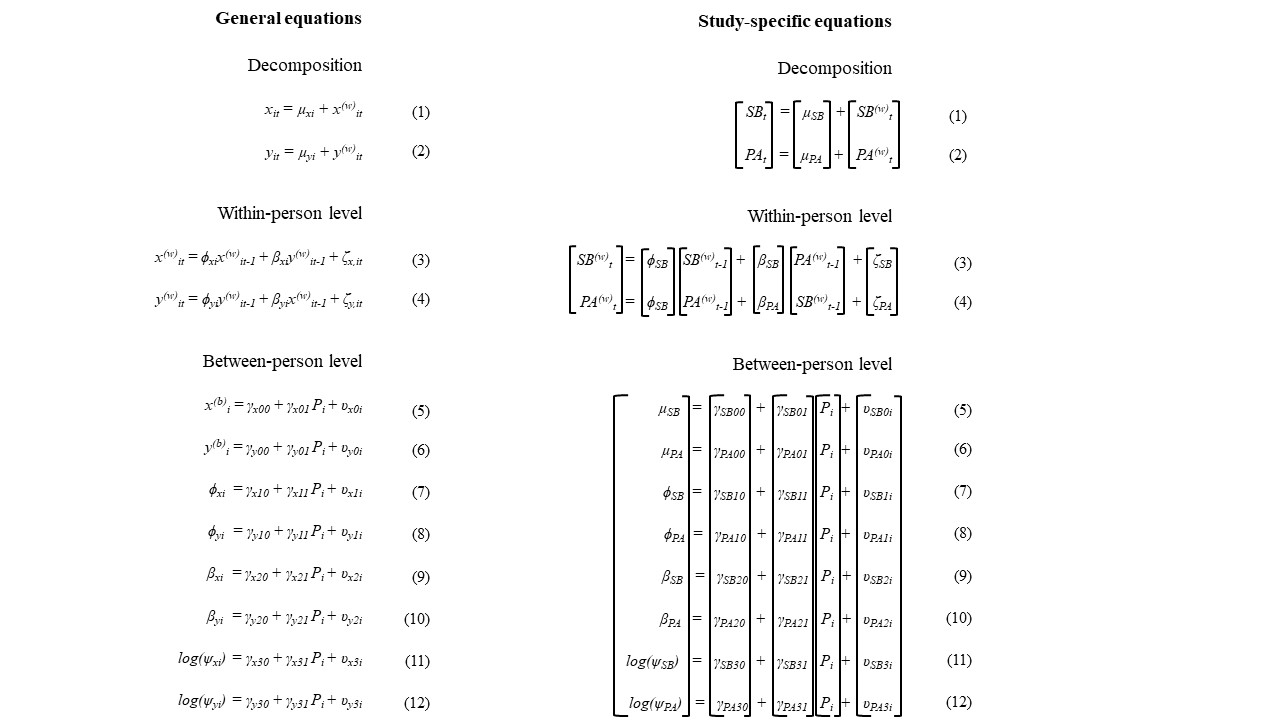


**Figure S1**. Representation of the general and study-specific equations of multilevel time series dynamic structural equation models.

**Notes**. The equations show the decomposition of the data into within- and between-person level, including an observed predictor for random effects at the between level. Study-specific equations: The symbols correspond to the symbols used in *Figure 1*.

**Abbreviations**. SB = sedentary behavior, PA = physical activity. General equations: Decomposition: _i_ = individuals, _t_ = time points, *x* = observed variable, *y* = observed variable, µ = between-person means, ^(w)^ = within-person estimates. Within-person level: _i_ = individuals, _t_ = time points, _t-1_ = previous time points, *x* = observed variable, *y* = observed variable, ϕ = autoregressive estimates, β = bidirectional (cross-lagged) estimates, ζ = within-person, time-specific residuals. Between-person level: ^(b)^ = between-person estimates, _i_ = individuals, _t_ = time points, *x* = observed variable, *y* = observed variable, µ = between-person means, φ = autoregressive estimates, β = bidirectional (cross-lagged) estimates, ψ = dynamic errors, υ = between level residuals, P = predictor.


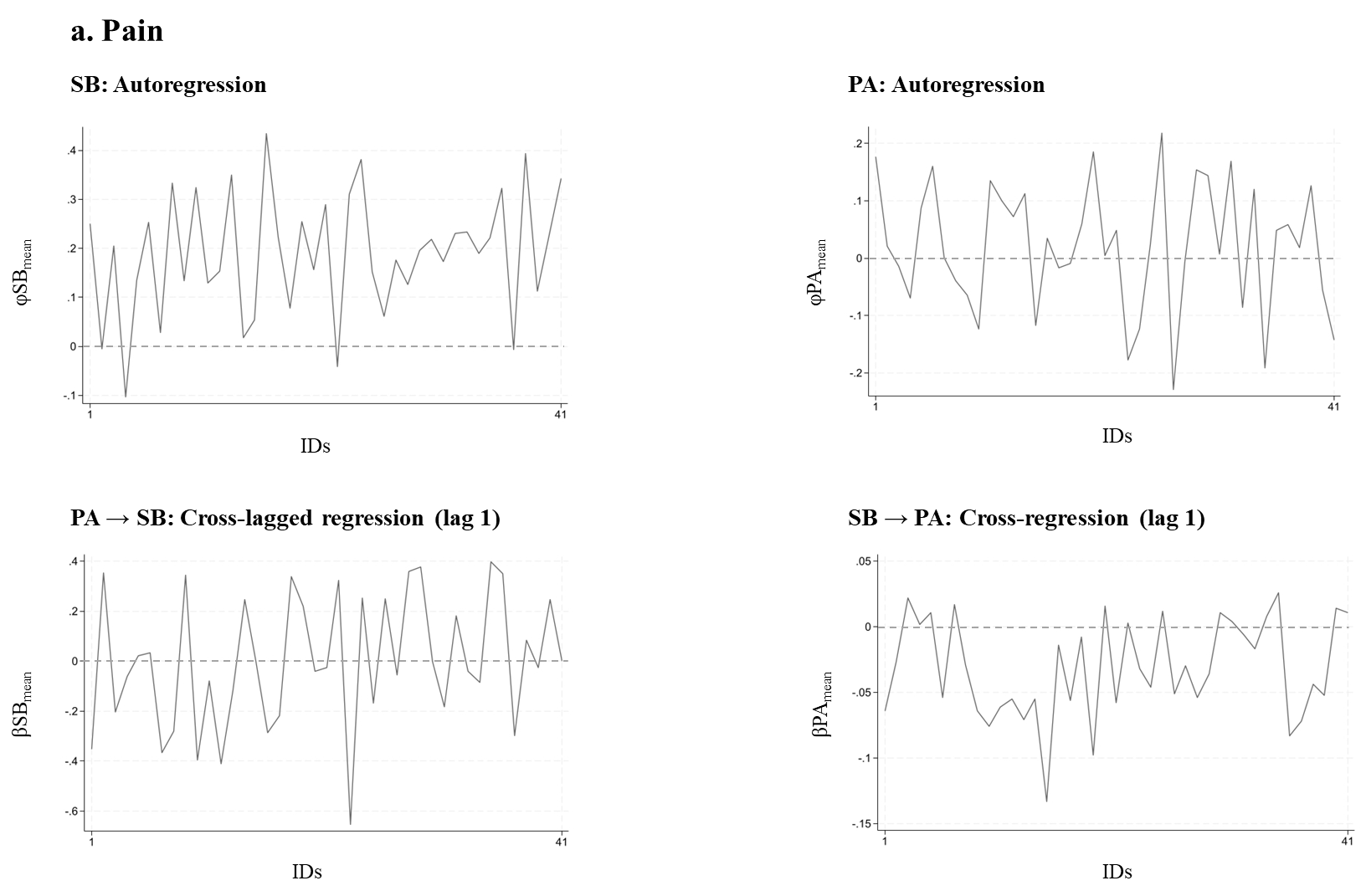


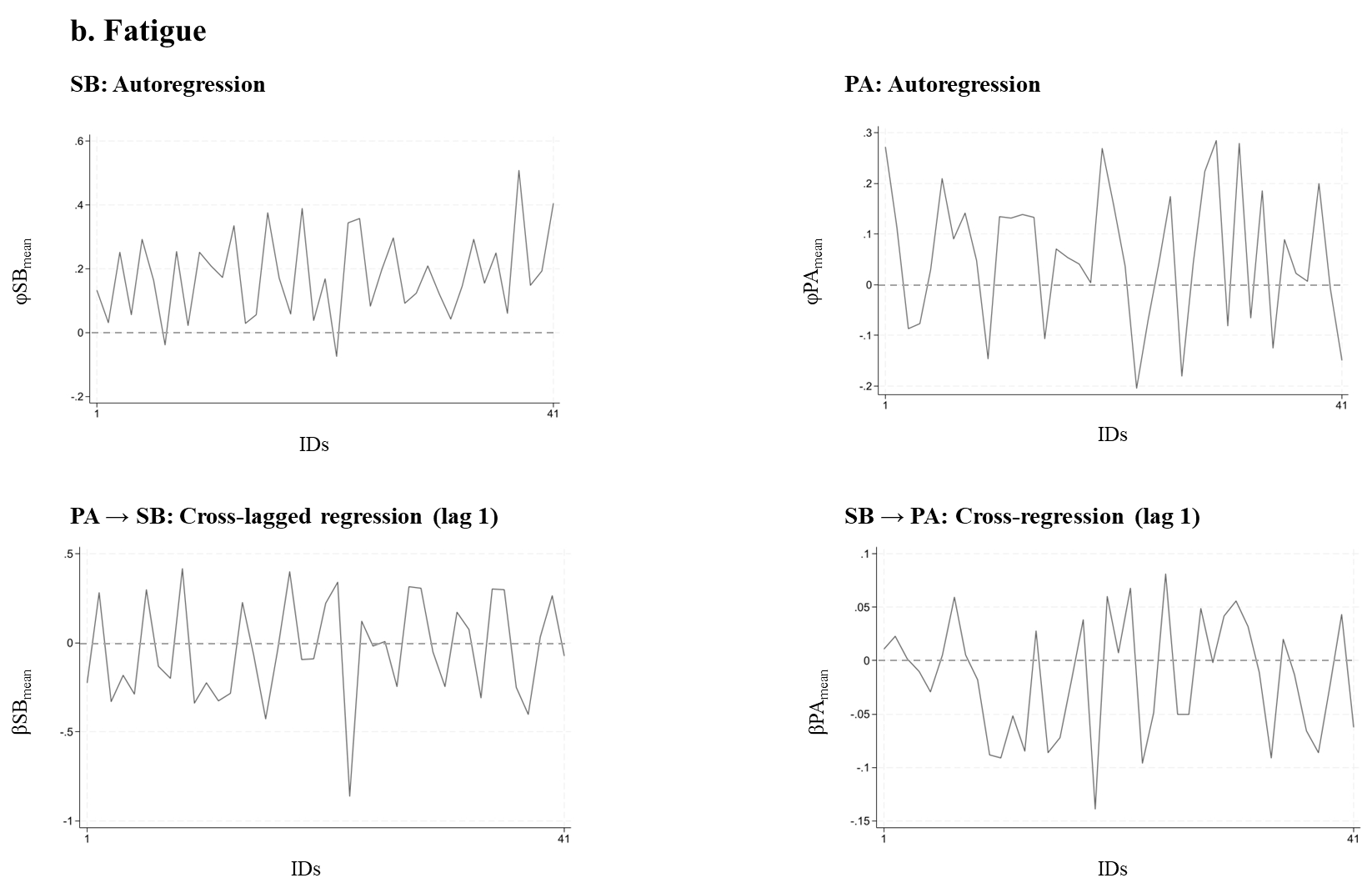


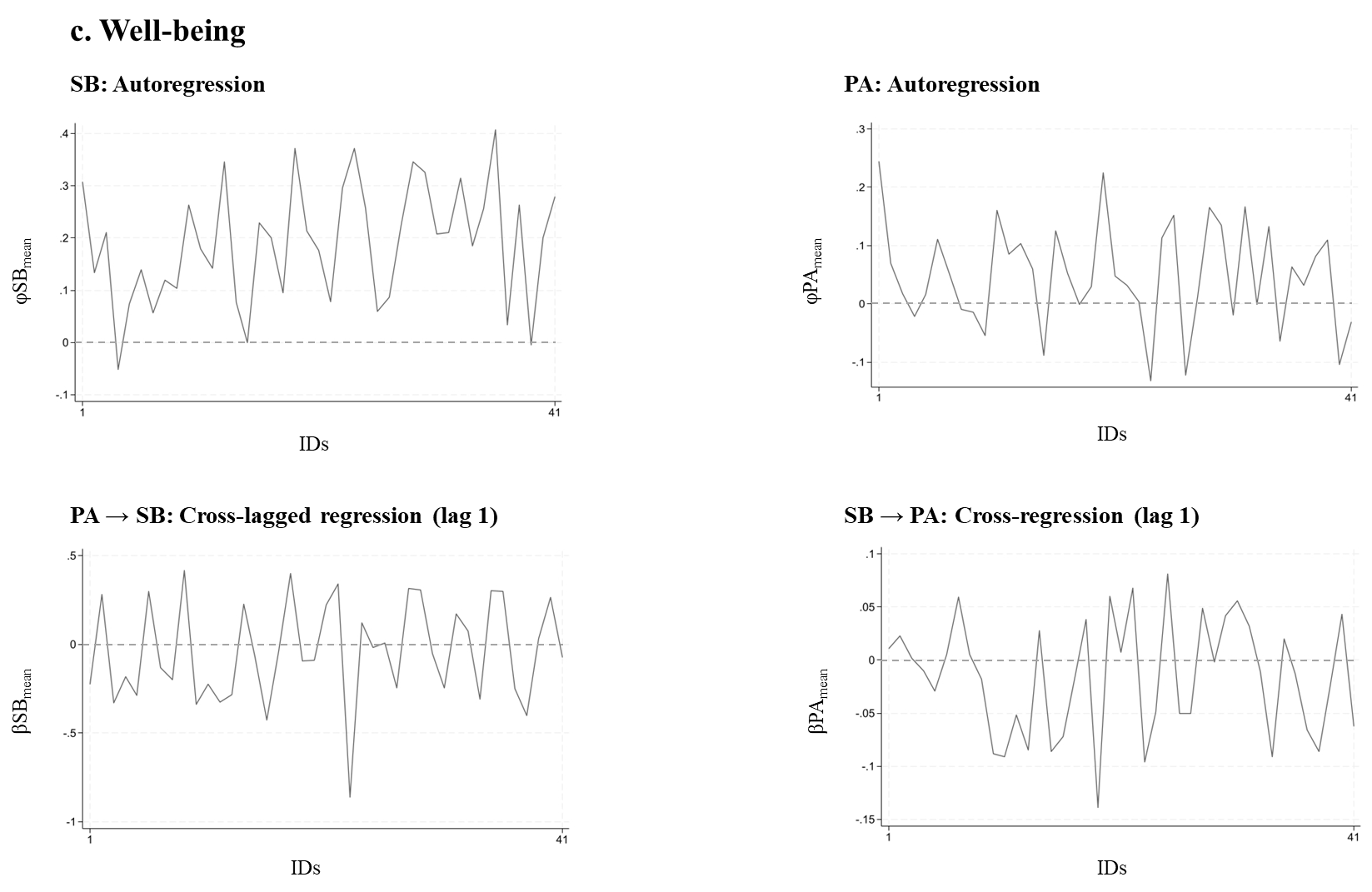


**Figure S2**. Results of individual's absolute standardized autoregressive and cross-lagged regression coefficients for the average time spent in sedentary behavior or physical activity for the three DSEMs.

**Notes**. (a) Results for pain. (b) Results for fatigue. (c) Results for well-being. To illustrate the results, a grey dashed line has been inserted to indicate the value zero; i.e., the “typical” amount of time an individual spends in SB or PA.

**Abbreviations**. SB = sedentary behavior, PA = physical activity, ϕ = autoregressive estimates, β = bidirectional (cross-lagged) estimates.
